# Mathematical Modeling of Patient Care

**DOI:** 10.1155/2012/563287

**Published:** 2012-05-28

**Authors:** Philip Crooke, John Hotchkiss, Yongwimon Lenbury, Brett McKinney

**Affiliations:** ^1^Department of Mathematics, Vanderbilt University, Nashville, TN, USA; ^2^Department of Critical Care Medicine and Department of Medicine, Pittsburgh Veterans Affairs Healthcare System, University of Pittsburgh Medical Center, Pittsburgh, PA, USA; ^3^Department of Mathematics, Faculty of Science, Centre of Excellence in Mathematics (CHE), Mahidol University, Bangkok 10400, Thailand; ^4^Tandy School of Computer Science and Department of Mathematics, Rayzor Hall, University of Tulsa, Tulsa, OK 74104, USA

In our call for papers for this special issue, we requested topics that ranged from mathematical models of physiological systems to macroeconomic models of the healthcare industry. Although the response to this broad invitation was muted, we did receive a nice cross-section of papers within this spectrum of topics. In the following, we summarize the six papers included in this volume.

D. T. Grima et al. investigated the impact of antibiotics on treating *Clostridium difficile*-associated diseases (CDAD) on the emergence of antibiotic-resistant bacteria using mathematical model describing the impact of in-hospital antibiotic use. The model is a system of nonlinear differential equations arising from a compartment network of uncolonized and colonized patients with and without antibiotic treatment. The model leads to a very important conclusion: there may be a substantial reduction in antibiotic-resistant prevalence when nonantibiotic strategies are employed for CDAD. This is a perfect example of how mathematical modeling can lead to a practical change in treatment and impact on patient health and a reduction in costs.

Advances in mathematical modeling have led to various effective tools for early diagnosis, such as the application of thermometry ultrasonic techniques. Before implementation of the ultrasonic noninvasive estimation of thermal gradients into tissues based on spectral changes, rigorous analyses, over transient echotraces acquired from well-controlled biological and computational phantoms, must be still made in order to improve resolutions and evaluate clinic limitations. I. Bazan et al. applied this technique to computationally modeled echotraces from scattered biological phantoms, attaining high resolution (better than 0.1°C). They also provided computer methods for viability evaluation of thermal estimation from echoes with distinct noise levels, making it difficult to interpret the readings. As a result of their analyses, the technique has been evaluated as a possible effective diagnosis tool for scattered tissues applications. Such research and analyses are of great importance since the ability to detect changes of thermal origin in ultrasonic echo spectra means the achievement of precise noninvasive temperature estimations that could be very useful as an early complementary indicator of infections, inflammations, or cancer.

Obstructive sleep apnea (OSA) is an important health issue and has been subject to different modeling investigations. C. T. Su et al. have applied a Mutliclass Mahalanobis-Taguchi System (MMTS) to the problem of prediagnosing the condition using readily available patient information. Their model results in a better than 84% accuracy. This statistical diagnostic/predictive method comes from pattern information technology and demonstrates an important application of a little used modeling paradigm in biology.

The paper by C. Pagel et al. addresses a problem of growing relevance with an aging population, dwindling resources, and a declining number of healthcare providers: that of optimal scheduling in the context of a stepped care. This topic is of considerable interest, not only in the domain addressed by the authors, but in other populations requiring complex, highly specialized care. The author's model includes stratified levels of intensity of care, and can be used to analyze expected changes in waiting times and throughput under different allocations of scheduled slots. This quantitative model will be of highly operational interest—particularly to large, integrated healthcare systems confronted with a growing mental healthcare population. Moreover, since the model is sufficiently flexible, it may be adapted to address queuing systems germane to other highly complex medical conditions.

Healthcare providers and networks face more than resource limitations: increased oversight and an emphasis on the quality of care are wending their way toward reimbursement (“pay for performance”) as well as public access to quality “report cards.” In this context, establishing that the metrics of quality accurately reflect the performance of the hospital, rather than differences in patient populations, is of paramount importance. The paper by D. Shine directly addresses this issue, focusing on the modulation of risk by comorbid conditions. Shine illustrates a spreadsheet-based approach for capturing high impact comorbidities and identifies those having the greatest impact on risk. This general approach could improve the accuracy of expected mortality estimates, thus also the ratio of observed mortality to expected mortality. Such considerations will have a clear impact not only on quality reports but also eventually on equitable reimbursement.

HIV/AIDS is a global pandemic of disastrous proportions, in which the virus can rapidly develop resistance to antiretrovirals, and subsequently, lifelong therapy is required, and many at-risk individuals live in austere environments where compliance with complex treatment regimens may prove problematic. These medications are often toxic, and attenuation of treatment intensity due either to toxicity or to regimen complexity can promote the development of drug resistance. The development of new antiretroviral agents or formulations is both costly and critical in the setting of a virus prone to very rapid mutation. M. L. Branham et al. illustrate the use of artificial neural networks (ANN), primed with drug physicochemical and bioaccumulation characteristics, to predict maximum recommended therapeutic doses of antiretroviral agents. They note particular utility of the ANN approach when the medications evaluated are poorly soluble in water. This type of computational analyses should prove useful as drug developers balance bioavailability and ease of administration against tolerable doses, a balance with implications for compliance and the potential for effective treatment.

We are convinced of the utility of mathematics and other quantitative approaches to provide better healthcare. The six papers in this volume demonstrate that mathematical modeling can play a central role in patient care innovations.

*Philip Crooke* *John Hotchkiss* *Yongwimon Lenbury* *Brett McKinney*

